# Cell Type-Specific Metabolic Response to Amino Acid Starvation Dictates the Role of Sestrin2 in Regulation of mTORC1

**DOI:** 10.3390/cells11233863

**Published:** 2022-11-30

**Authors:** Biljana Blagojevic, Fadi Almouhanna, Gernot Poschet, Stefan Wölfl

**Affiliations:** 1 Institute of Pharmacy and Molecular Biotechnology, Heidelberg University, Im Neuenheimer Feld 364, 69120 Heidelberg, Germany; 2 Metabolomics Core Technology Platform, Centre for Organismal Studies, Heidelberg University, Im Neuenheimer Feld 360, 69120 Heidelberg, Germany

**Keywords:** Sestrin2, amino acid deprivation, mTORC1, nutritional stress, metabolic adaptation

## Abstract

Targeting cancer metabolism has become one of the strategies for a rational anti-tumor therapy. However, cellular plasticity, driven by a major regulator of cellular growth and metabolism, mTORC1, often leads toward treatment resistance. Sestrin2, a stress-inducible protein, has been described as an mTORC1 inhibitor upon various types of stress signals. Immune assays and online measurements of cellular bioenergetics were employed to investigate the nature of Sestrin2 regulation, and finally, by silencing the SESN2 gene, to identify the role of induced Sestrin2 upon a single amino acid deprivation in cancer cells of various origins. Our data suggest that a complex interplay of either oxidative, energetic, nutritional stress, or in combination, play a role in Sestrin2 regulation upon single amino acid deprivation. Therefore, cellular metabolic background and sequential metabolic response dictate Sestrin2 expression in the absence of an amino acid. While deprivations of essential amino acids uniformly induce Sestrin2 levels, non-essential amino acids regulate Sestrin2 differently, drawing a characteristic Sestrin2 expression fingerprint, which could serve as a first indication of the underlying cellular vulnerability. Finally, we show that canonical GCN2-ATF4-mediated Sestrin2 induction leads to mTORC1 inhibition only in amino acid auxotroph cells, where the amino acid cannot be replenished by metabolic reprogramming.

## 1. Introduction

Warburg’s discovery that cancer cells elevate aerobic glycolysis, and Farber’s anti-folate treatment of childhood lymphocytic leukemia opened a new era of anticancer treatment—targeting cancer metabolism [1]. Extensive research revealed several metabolic vulnerabilities in cancer cells, i.e., lactate production [2,3] or amino acid metabolism [4,5]. However, until now, there are only few approved metabolic modulators, either due to lack of specificity, or due to metabolic similarities between highly proliferative, cancer and immune, cells [6]. Therefore, a more systematic approach is needed to identify a tissue, or even lineage-specific metabolic features for more precise targeting. 

mTORC1, the mechanistic target of rapamycin, mTORC1 is a major regulator of cellular growth and metabolism, as a response to growth signal and nutrient availability [7]. Hyperactive mTORC1 in cancer cells leads to overall increase in anabolic and reduction in catabolic reactions to support cellular proliferation [8]. Small GTPases are channeling the signals coming from growth factors (RHEB) and nutrient availability (Rags), which altogether leads to mTORC1 lysosomal translocation and sequential activation [7,9]. Understanding of mTORC1 regulation is necessary for rational mTORC1 targeting [9,10,11]. 

A negative regulator of mTORC1 activity in response to various types of stress is Sestrin2, the most investigated member of the sestrin protein family [12,13]. Sestrin2 is a stress-inducible protein, regulated by DNA damage, oxidative, ER, nutritional stress, and others [14,15,16,17,18,19]. Although initially discovered as a p53-inducible gene [16], other transcription factors, such as ATF4 [20] and C/EBPβ [19], have been linked to induction of SESN2 gene expression. Nutritional stress, caused by amino acid removal, activates the GCN2-ATF4 axis and downstream induces Sestrin2 expression to inhibit mTORC1 [20]. Although reported as a leucine sensor for mTORC1 [21], Sestrin2 regulates mTORC1 in the absence of lysine, glutamine, or arginine [20,22].

Due to sensitivity to stress signals, we sought to investigate the underlying context that leads to Sestrin2 regulation upon a single amino acid deprivation. We identified a complex interplay of various types of stress that regulates Sestrin2. Importantly, we identify a cell-line-dependent Sestrin2 regulation upon amino acid deprivation revealing an underlying metabolic vulnerability of the cell line. Finally, we showed the importance of Sestrin2 regulation to modulate mTORC1 activity, which becomes essential/more important in amino acid auxotroph cells.

## 2. Materials and Methods

### 2.1. Cell Culture

Cell lines used in this project: HT29, HCT116, HCT116 p53-/-, HepG2, HLE, SKHep, MCF7, MDA-MB-231, MDA-MB-468, T47D, LnCap, and Du145, were cultured in standard cell culture incubator, at 37 °C, 5% CO_2_, in Dulbecco’s modified Eagle medium—DMEM + GlutaMAX (Gibco, Carlsbad, CA, USA, via Thermo Fischer Scientific GmbH, Dreieich, Germany). Media was supplemented with 10% FCS (*v*/*v*) (Gibco, Carlsbad, CA, USA, via Thermo Fischer Scientific GmbH, Dreieich, Germany) and 1% penicillin/streptomycin (*v*/*v*) (Gibco, Carlsbad, CA, USA, via Thermo Fischer Scientific GmbH, Dreieich, Germany). MDA-MB-468 and T47D cells were a kind gift from Dr. Stefan Wiemann (DKFZ, Heidelberg, Germany). HCT116 p53/- were a kind gift from Dr. Thomas G. Hofmann (DKFZ, Heidelberg, Germany) [23]. Single amino acid-deficient media were prepared following the DMEM + GlutaMAX recipe, by omitting the indicated amino acid. All deprived media, including self-prepared replete medium, were supplemented with dialyzed FCS (10%) and penicillin/streptomycin (1%). Short incubation times (up to 9 h) were performed by conditioning cells with full medium supplemented with dialyzed FCS, a day prior to the treatment. For longer treatments (longer than 24 h), medium was directly exchanged with replete/deprived medium. Additional treatments were added to the medium and incubated as described in the figure legends. N-acetylcysteine was purchased from Sigma Aldrich (Taufkirchen, Germany), sodium formate from Carl Roth GmbH (Karlsruhe, Germany), and GCN2 inhibitor (GZD-824) and PERK inhibitor (GSK2656157) from Cayman Chemical (Ann Arbor, USA; via Biomol GmbH, Hamburg, Germany). 

### 2.2. Western Blot

Cells were seeded in a 6-well plate at the density of 200,000–300,000 cells/well. The following day cells were treated with deprived media for longer incubation times, or with full medium (supplemented with dialyzed FCS) for shorter time points, and finally, 1–9-h treatments were performed the day later. At indicated time-points, cells were lysed with 6 M urea buffer supplemented with protease and phosphatase inhibitors (10 µg/mL pepstatin, 0.1 µg/mL PMSF, 10 µg/mL aprotinin, 2.5 mM sodium pyrophosphate, 1 mM sodium orthovanadate). Total protein content was measured using Bradford reagent (Sigma Aldrich, Taufkirchen, Germany) and equal protein amounts (20 µg for Sestrin2; 40 µg for other antibodies) were loaded on 10% SDS-PAGE. After separation, proteins were transferred onto a PVDF membrane (GE Healthcare, Munich, Germany). Ponceau S (Sigma Aldrich, Taufkirchen, Germany) was used to validate uniform transfer. After blocking in 5% milk (*w*/*v*) for at least 1.5 h at room temperature, membranes were incubated with respective primary antibodies overnight, at 4 °C. Finally, anti-mouse or anti-rabbit IgG horseradish peroxidase (HRP)-linked secondary antibody (Cell Signaling Technology Europe, Frankfurt am Main, Germany) was prepared in 5% milk and used to incubate membranes for 1 h at room temperature. Membranes were developed with ECL (100 Mm Tris/HCL pH 8.6, 2.1 Luminol, 0.6 mM p-Coumaric acid, 5.3 mM hydrogen peroxide) and imaged with Fujifilm LAS-3000 (Fujifilm, Germany).

Primary antibodies used in this study: Sestrin2 (Proteintech, Planegg-Martinsfried, Germany), total AMPKα, total p38, Actin, Vinculin (Santa Cruz Inc, Heidelberg Germany), phospho-P70S6K (R&D Systems, McKinley Place NE Minneapolis, USA), phospho-AMPKα, total P70S6K, ATF4, and phospho-p38 (Cell Signaling Technology Europe, Frankfurt am Main, Germany).

### 2.3. Gene Silencing

SESN2 silencing was performed using a pool of small interference RNA targeting SESN2 purchased from Riboxx (Riboxx Pharmaceuticals, Dresden, Germany): AUAAUCUAAUACUCCCUCCCCC, UUACAGUCAUCACAUGCCCCC, UUUAUGUACUUGGCCUCUCCCCC. The following sequence was used as a negative control (siNC): UUGUACUACACAAAAGUACCCCC. Gene silencing was done using reverse transfection method: fifty picomoles of respective siRNA were precomplexed with 1 µL of Lipofectamine 3000 (Thermo Fisher Scientific GmbH, Dreieich, Germany) in 50 µL of OptiMEM medium (Gibco, Carlsbad, CA, USA, via Thermo Fischer Scientific GmbH, Dreieich, Germany) and added to 100,000 cells in 1 mL of DMEM + GlutaMAX (Gibco, Carlsbad, CA, USA, via Thermo Fischer Scientific GmbH, Dreieich, Germany). Cells were then seeded in a 6-well plate and treated on the following day for the indicated treatments. Proteins of interest were analyzed with Western blot, as described above.

### 2.4. Protein Synthesis

Puromycin incorporation was used to study protein synthesis in the cells with lower expression of Sestrin2. The SESN2 gene was silenced using reverse transfection, as described before. On the following day, medium was exchanged to either full, or serine-deficient medium for 24 h treatment. Five hours prior to lysis, indicated cells were treated with 20 µM cycloheximide, CHX, (Fluka Sigma Aldrich, Taufkirchen, Germany). Finally, 10 min before lysis cells were treated with 90 µM of puromycin (Thermo Fisher Scientific GmbH, Dreieich, Germany). Cells were then lysed and the Western blot was performed as described.

### 2.5. Cell Survival

Cellular survival was measured with Sulforhodamine B (SRB) assay (Santa Cruz Biotechnology Inc, Heidelberg, Germany). Cells were seeded in a 96-well plate at the density of 5000–7000 cells/well. Media lacking single amino acid was added on the following day. At the indicated time-points, plates were fixed with ice-cold 10% trichloroacetic acid (TCA) and stored at 4 °C for at least one hour. Plates were then washed with tap water and dried for 10 min. SRB dye (0.054% in acetic acid) was used to stain total protein for 30 min at room temperature. Dye excess was removed with 1% acetic acid and finally dissolved in 10 mM Tris (pH 10.5). Absorbance (535 nm) was measured with a Tecan Ultra plate reader (Tecan Deutschland GmbH, Crailsheim, Germany). Survival (%) was calculated in comparison to the averaged replicates with full medium.

### 2.6. ROS Formation Assay

Cytoplasmic superoxide was measured using dihydroethidium (DHE) (Cayman Chemical (Ann Arbor, USA; via Biomol GmbH, Hamburg, Germany). Cells were seeded in a 24-well plate at the density of 50,000 cells/well. At the indicated time-points, cells were washed with PBS (Gibco, Germany), and trypsinized with TriPLE Express (Gibco, Carlsbad, CA, USA, via Thermo Fischer Scientific GmbH, Dreieich, Germany). Collected cells were then incubated with 30 µM DHE dye for 10 min at room temperature, in the dark. The dye was removed by centrifugation and the cells were resuspended in colorless DMEM (Gibco, Carlsbad, CA, USA, via Thermo Fischer Scientific GmbH, Dreieich, Germany). Data acquisition was performed with Guava easyCyte HT (Luminex, MV ‘s-Hertogenbosch, The Netherlands), and analyzed with GuavaSoft 3.1.1.

### 2.7. Mitochondrial Membrane Potential

To measure mitochondrial membrane potential, cells were collected after indicated treatments using Trypsin TriPLE (Gibco, Carlsbad, CA, USA, via Thermo Fischer Scientific GmbH, Dreieich, Germany). Collected cells were then incubated with 18 µM JC-1 (Sigma Aldrich, Taufkirchen, Germany) for 30 min at room temperature, in the dark. Cells were then centrifuged and resuspended in fresh, colorless DMEM (Gibco, Carlsbad, CA, USA, via Thermo Fischer Scientific GmbH, Dreieich, Germany) for measurement, performed with Guava easyCyte (Luminex, MV ‘s-Hertogenbosch, The Netherlands). Acquired data were analyzed with GuavaSoft 3.1.1. 

### 2.8. Cellular Bioenergetics-Extracellular Acidification and Oxygen Consumption

Extracellular pH and oxygen were measured using PreSens SensorDish readers—SDR (PreSens, Precision Sensing GmbH, Regensburg, Germany). As described previously [24], cells were seeded at the density of 100,000 cells/well in a Hydro- or OxoDish 24-well plate for pH or oxygen measurements, respectively, and placed in a standard incubator. Treatments were added on the following day and the plates were placed onto the readers located inside the standard incubator. Measurements, obtained from sensors located at the bottom of each well, were acquired every 10 min. For statistical significance, slopes representing the consumption rate were calculated using linear regression from the initial linear range after stabilization of the readings.

### 2.9. Amino Acids Analysis

Free amino acids were extracted from 3 × 10^6^ cells with 0.3 mL of 0.1 M HCl in an ultrasonic ice-bath for 10 min. The resulting extracts were centrifuged for 10 min at 4 °C and 16,400× *g* to remove cell debris. Amino acids were derivatized with AccQ-Tag reagent (Waters, Eschborn, Germany) and determined as described in Weger et al. [25]. 

### 2.10. Statistical Analysis

Data were analyzed using Microsoft Excel or GraphPad Prism. The mean value was presented with error bars representing SEM. If not indicated otherwise, Student’s *t*-test adaptation, Welch’s *t*-test was used to calculate the difference between two groups. Statistical significance is represented with asterisks: *, **, ***, and ****, for values ≤ 0.05, 0.01, 0.001, and 0.0001, respectively. n.s. was used to indicate statistical insignificance.

## 3. Results

### 3.1. Sestrin2 Regulation ‘Fingerprint’: Cell Line-Dependent Regulation of Sestrin2 under a Single Non-Essential Amino Acid Deprivation

**Figure 1:**
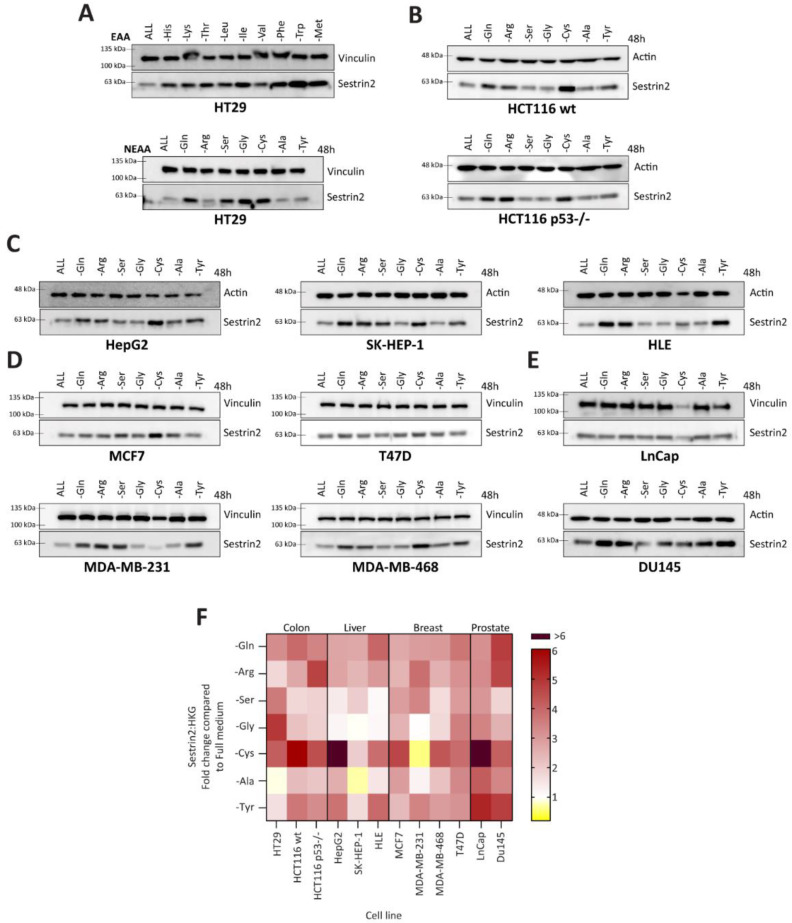
Sestrin2 regulation upon single amino acid deprivation. (**A**–**E**) Sestrin2 protein levels after two days of amino acid deprivations; (**F**) densitometric quantification of Western blots, Sestrin2 normalized to respective housekeeping gene. EAA—essential amino acids; NEAA—non-essential amino acids.

Firstly, we investigated Sestrin2 regulation upon nutritional stress caused by a single amino acid deprivation. Although deprivations of essential amino acids uniformly induced Sestrin2 protein levels, the absence of a single non-essential amino acid differently regulated Sestrin2 in HT29 cells (Figure 1A). Therefore, we screened cell lines with different tissue origins or genetic backgrounds for Sestrin2 expression under non-essential amino acid starvations (Figure 1B–E). Densitometric quantification of Western blots (Figure 1F) showed a cell line-, rather than an amino acid-dependent Sestrin2 regulation. Although cysteine deprivation induced Sestrin2 in almost all cell lines (except MDA-MB-231), serine deprivation differentially regulated Sestrin2 levels in the investigated cell lines. MCF7 and MDA-MB-231 cells that have lower expression of the rate-limiting enzyme in serine biosynthesis pathway, PHGDH [26] (Figure S1), and hence decreased serine biosynthesis [27], were dependent on an extracellular source of serine. Therefore, in MCF7 and MDA-MB-231 Sestrin2 protein levels were upregulated upon serine removal. On the other hand, in MDA-MB-468 and T47D cells that naturally have higher PHGDH level [26], Sestrin2 protein levels were not significantly changed. Removing single amino acids from the medium indicated a context-dependent nutritional stress. Moreover, Sestrin2, a stress marker, showed a cell line ‘fingerprint’ upon different amino acid deprivations.

**Figure 2:**
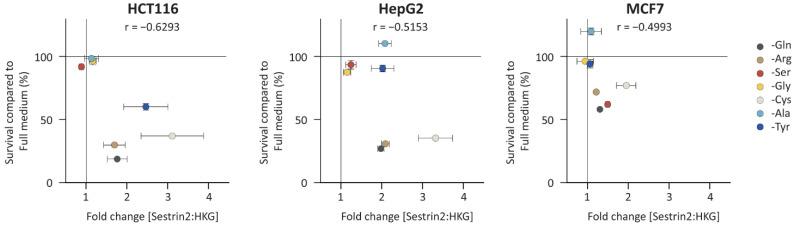
Negative correlation between Sestrin2 expression and cellular survival. Sestrin2 levels from three independent experiments were plotted on the xaxis; yaxis shows cellular survival measured with SRB assay. Pearson correlation was calculated and r value is indicated for each graph. *p* values are 0.13, 0.2366, and 0.2539 for HCT116, HepG2, and MCF7, respectively.

Being classified as a GADD (growth arrest and DNA damage) family member, SESN2 (formerly known as Hi95) was linked to cellular growth [16]. Therefore, we investigated a correlation between Sestrin2 expression and cellular survival under amino acid deprivation. Three cell lines from various tissue origins, namely, hepatocellular (HepG2), colorectal (HCT116), and breast (MCF7) cancer, that had different Sestrin2 fingerprints (Figure 1), were selected for further investigation. Figure 2 shows the correlation between Sestrin2 expression upon 48 h of amino acid deprivation with cellular survival 72 h after removing the same amino acid. As with Sestrin2 expression (Figure 1 and Figure S2A), the cellular survival was also differently regulated in the investigated cell lines upon the amino acid deprivations (Figure S2B). Interestingly, the reduced cellular survival was associated with higher expression of Sestrin2, i.e., glutamine, arginine, and cysteine deprivations in all investigated cell lines. However, several conditions, i.e., tyrosine deprivation in HepG2 cells increased Sestrin2 level, while cellular survival was not changed. Altogether, single amino acid deprivations differently regulated Sestrin2, which negatively correlated with cellular survival.

Of note, silencing SESN2 did not influence cellular proliferation upon amino acid starvation (Figure S2C). These data clearly indicate that Sestrin2 upregulation coincides with reduced survival, but it is dispensable for the proliferation regulation upon single amino acid starvation. Interestingly, silencing SESN2 severely reduced cellular survival, but only in MCF7 cells (Figure S2D).

Based on obtained data, the single amino acid deprivations were classified into three categories: first, glutamine, arginine, or cysteine deprivation, that induced Sestrin2 expression and inhibited cellular proliferation in all three investigated cell lines; second, serine (and glycine) deprivation that caused cell line-dependent response with the increased Sestrin2 and the reduced cell survival only in MCF7 cells; and third, alanine or tyrosine deprivation as outliers, that induced Sestrin2 regulation without changing cell survival in HepG2 cells. 

### 3.2. Glutamine, Arginine, or Cysteine Deprivation Causes Severe Cellular Response That Induces Sestrin2 via Oxidative, Metabolic, and Nutritional Stress 

**Figure 3:**
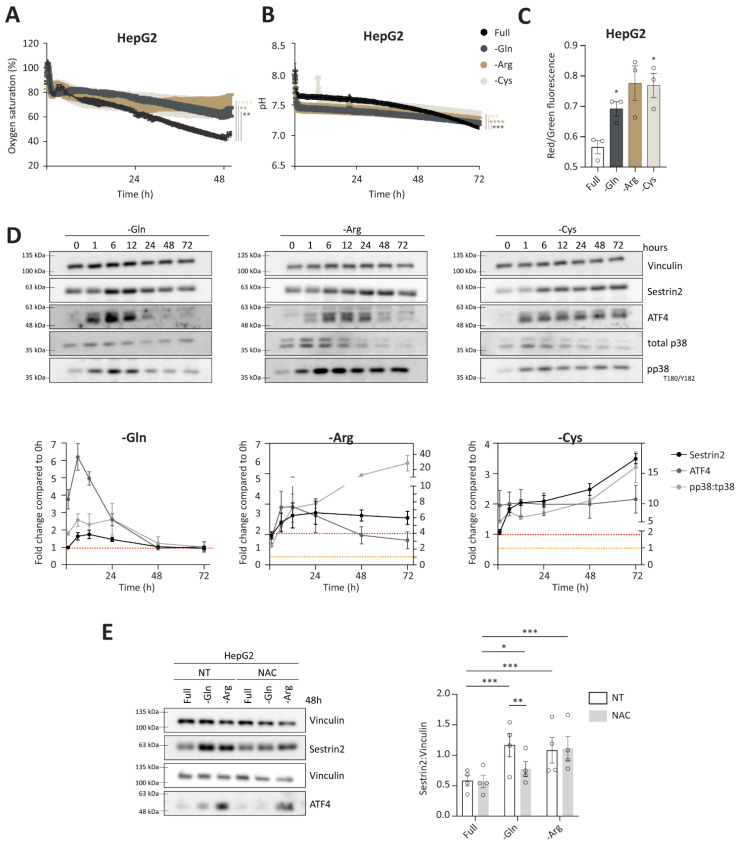
Group 1 of amino acid deprivations causes metabolic, oxidative, and nutritional stress. (**A**,**B**) Continuous measurements of oxygen saturation (**A**) and pH (**B**) values in culture medium. Two-way ANOVA with Dunnett’s post hoc test was used to calculate statistical significance between slopes; (**C**) mitochondrial membrane potential measured 48 h of amino acid deprivations; (**D**) time-dependent regulation of Sestrin2, ATF4, and phosphorylation of p38 upon the deprivations in HepG2 cells, with the densitometric quantification (n = 3). ATF4 and pp38:tp38 in arginine and cysteine deprivation correspond to the right yaxis. Red dashed line indicates fold change = 1 on the left yaxis, yellow dashed line indicates fold change = 1 on the right yaxis; (**E**) Sestrin2 and ATF4 levels upon 10 mM Nacetyl cysteine co-treatment with respective densitometric quantification (n = 4). Two-way ANOVA with Tukey’s post hoc test was used for statistical analysis. Dots represent individual values. *, **, ***, and **** indicate *p* ≤ 0.05, 0.01, 0.001, and 0.0001, respectively.

Having established a negative correlation between Sestrin2 expression and cellular survival in the absence of glutamine, arginine, or cysteine, we sought to understand the overall cellular response to the removal of glutamine, arginine, or cysteine, and to isolate events that were leading to Sestrin2 upregulation. Online measurements of extracellular oxygen saturation, reflecting oxygen consumption, and pH, reflecting glycolysis, showed an altered glucose metabolism under glutamine, arginine, or cysteine deprivations in HepG2 (Figure 3A,B) and HCT116 cells (Figure S3A,B). To test mitochondrial function, we measured mitochondrial membrane potential in group 1 of the amino acid deprivations. As shown in Figure 3C, increased mitochondrial membrane potential was observed after 48 h in HepG2 cells. The same was observed in HCT116 cells, while in MCF7 cells mitochondrial membrane potential was changed upon arginine deprivation solely (Figure S3C). Additionally, we followed phosphorylation of the MAPK kinase, p38 (T180/Y182), controlled by increased intracellular reactive oxygen species, DNA damage, or inflammation (here used as a marker of oxidative stress) [28]. Sestrin2 upregulation was accompanied by p38 (T180/Y182) phosphorylation under all three amino acid deprivations in HepG2 cells (Figure 3D). Increased mitochondrial membrane potential, together with phosphorylation of p38 suggested an elevated intracellular ROS generation. 

A canonical pathway to control amino acid availability is GCN2-ATF4 axis, hence, we followed the ATF4 expression. As shown in Figure 3D, ATF4 levels were increased upon amino acid removal, accompanying Sestrin2 upregulation. As ATF4 levels can also be induced upon oxidative stress, we cotreated cells with ROS scavenger, N-acetyl cysteine (NAC). Scavenging ROS rescued ATF4 and partially Sestrin2 levels upon glutamine deprivation. (Figure 3E and Figure S3D). Interestingly, the magnitude of Sestrin2 induction in the absence of glutamine or arginine was not aligned with the intensity of ATF4 regulation, until ROS was scavenged. 

Altogether, we showed that glutamine, arginine, and cysteine deprivations uniformly induced Sestrin2 in all investigated cell lines. The induction was mediated by at least two different mechanisms, through upregulated ROS levels and through the absence of the amino acid. Interestingly, when ROS levels were scavenged with NAC, Sestrin2 levels were regulated through ATF4. These data show a multilayered cellular regulation of Sestrin2 in response to amino acid deprivation.

Of note, an early regulation in phosphorylation of p38 and ATF4, observed up to 6 h of deprivation, was also noticed in the full medium (Figure S3E), therefore, in this study we focused on long-term effects of amino acid deprivations.

### 3.3. Sestrin2 Is Induced through Canonical GCN2-ATF4 Axis in Serine Auxotroph, MCF7 

**Figure 4:**
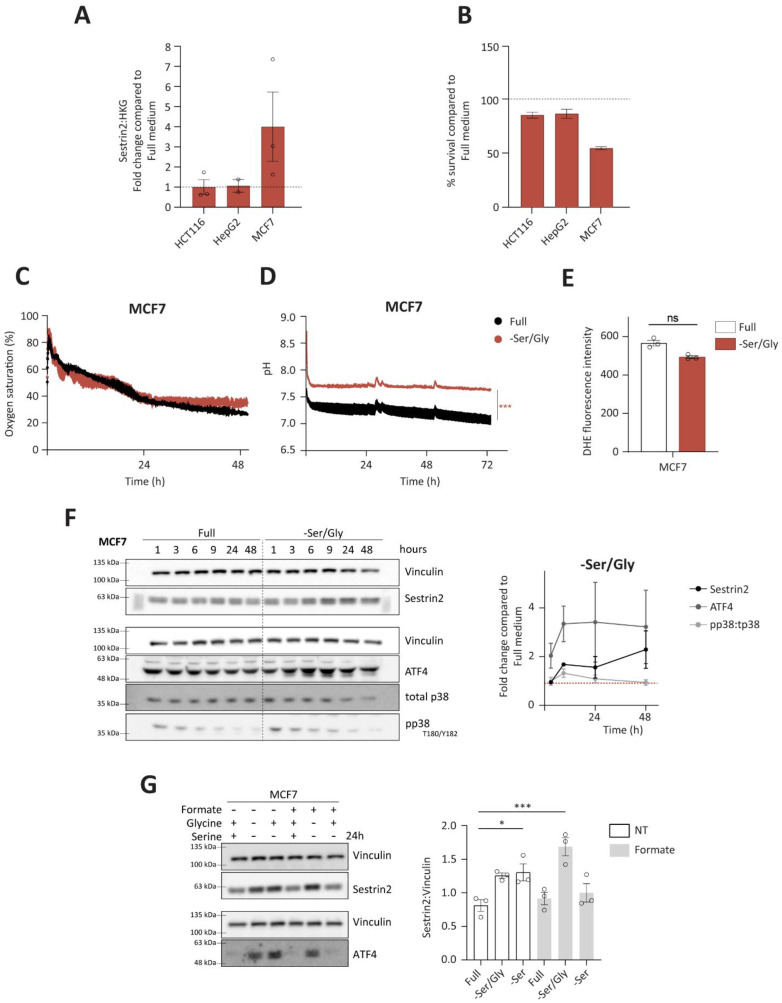
Group 2 of amino acid deprivations causes solely nutritional stress. (**A**) Quantification of Sestrin2 protein levels upon 2 days of serine and glycine deprivation (n = 3); (**B**) cell survival upon 3 days of serine and glycine deprivation; (**C**,**D**) continuous measurements of oxygen saturation (**C**) and pH (**D**) values in extracellular medium; (**E**) mitochondrial membrane potential measured 24 h of amino acid deprivations; (**F**) timedependent regulation of Sestrin2, ATF4, and phosphorylation of p38 upon the deprivation with respective densitometric quantification (n = 2–3); (**G**) Sestrin2 and ATF4 levels in the presence or absence of either serine (0.4 mM), glycine (0.4 mM), sodium formate (1 mM), or in combination, with the densitometric quantification of Sestrin2 (n = 3). One-way ANOVA with the Dunnett’s post hoc test was for statistical analysis. Dots represent individual values. * and *** indicate *p* ≤ 0.05, and 0.001, respectively. n.s. indicates statistical insignificance.

Due to metabolic interchangeability of serine and glycine, we firstly investigated Sestrin2 expression (Figure 4A), and cellular survival (Figure 4B) upon combined serine and glycine removal in all investigated cell lines. Again, only MCF7 cells were sensitive to the deprivation, hence they were used for further investigation of the Sestrin2 regulation in this biological context. Interestingly, metabolic analysis of intracellular amino acid levels revealed rather comparable reduction of serine and glycine levels in HepG2 and MCF7 cells indicating that differential regulation of Sestrin2 in these two cell lines is rather a downstream effect of reduced availability of these two amino acids (Figure S4A,B). As shown in Figure 4C,D, only mild changes in respiration and acidification rates were observed at later time-points in MCF7 cells. HCT116 cells did not show the change in their respiration and acidification rates (Figure S4C,D). Furthermore, no further accumulation of intracellular ROS (Figure 4E), accompanied with unchanged phosphorylation of p38 over time (Figure 4F), was observed in MCF7 cells upon serine/glycine deprivation. Additionally, neither ROS (Figure S4E), nor mitochondrial membrane potential (Figure S4F), was significantly increased in the investigated cell lines upon serine removal. Altogether, these data show the absence of oxidative stress upon serine/glycine deprivation in the investigated cells.

Furthermore, ATF4 levels were induced (Figure 4F) in MCF7 cells, unlike in HCT116 and HepG2 cells (Figure S4G). Finally, restoring one carbon cycle by adding formate and glycine, and sequentially replenishing serine levels [29], rescued ATF4 and Sestrin2 levels, indicating that solely absence of serine was responsible for its induction (Figure 4G). 

To conclude, unlike group 1 of amino acid deprivations that regulated Sestrin2 through complex interplay of nutritional, metabolic, and oxidative stress in all investigated cell lines, group 2 showed a cell line dependent regulation of Sestrin2 driven solely by nutritional stress. In serine auxotroph cells, serine/glycine deprivation impaired cellular survival without substantial metabolic reprogramming. Hence, induced Sestrin2 levels in the absence of serine/glycine were mainly the result of activated GCN2-ATF4 axis.

### 3.4. Alanine or Tyrosine Deprivation Regulates Sestrin2 Expression Solely through Oxidative Stress

**Figure 5:**
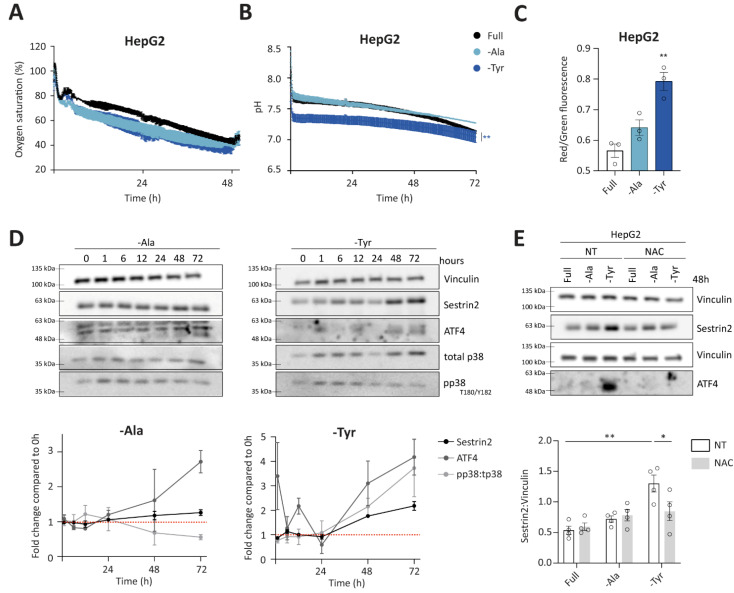
Group 3 of amino acid deprivations causes oxidative stress. (**A**,**B**) Continuous measurements of oxygen saturation (**A**) and pH (**B**) values in extracellular medium. Twoway ANOVA with Dunnett’s post hoc test was used to calculate statistical significance between slopes; (**C**) mitochondrial membrane potential measured 48 h of amino acid deprivations; (**D**) time-dependent regulation of Sestrin2, ATF4, and phosphorylation of p38 upon the deprivations in HepG2 cells, with respective densitometric quantification (n = 3); (**E**) Sestrin2 and ATF4 levels upon 10 mM Nacetyl cysteine cotreatment with densitometric quantification (n = 3). Twoway ANOVA with Tukey’s post hoc test was used to calculate statistical significance. Dots represent individual values. * and ** indicate *p* ≤ 0.05 and 0.01, respectively.

Group 3 consists of outliers from the negative correlation between Sestrin2 expression and cellular survival. In the absence of alanine or tyrosine, oxygen saturation and pH were comparable to the full medium in HepG2 and HCT116 cells (Figure 5A,B and Figure S5A,B). A trend towards increased mitochondrial membrane potential in deprived conditions was observed in all investigated cell lines, however it was significant only under tyrosine deprivation (Figure 5C and Figure S5C). Similarly, prolonged tyrosine deprivation, but not alanine deprivation, increased the phosphorylation of p38 in HepG2 cells (Figure 5D). Altogether, increased mitochondrial membrane potential and phosphorylation of p38 suggested a superoxide generation and subsequent oxidative stress upon tyrosine deprivation. Finally, ROS scavenger rescued both ATF4 and Sestrin2 levels upon tyrosine deprivation in both, HepG2 and HCT116 cells (Figure 5E and Figure S5D, respectively), indicating that solely oxidative stress triggered Sestrin2 upregulation under this condition. Alanine deprivation-induced Sestrin2 was partially rescued in HCT116 cells (Figure S5D). 

Interestingly, unlike the first two groups, group 3 did not follow the negative correlation between Sestrin2 protein levels and cellular survival. Tyrosine deprivation is a clear example that a single amino acid restriction can cause metabolic reprogramming and result in a mild oxidative stress, hence inducing Sestrin2 levels in HepG2 and HCT116 cells, while having minor (in HCT116) or no (in HepG2) impact on cellular proliferation. While metabolic background probably again plays a role in Sestrin2 regulation, this group of amino acid deprivation shows us that solely oxidative stress could regulate Sestrin2 upon an amino acid removal.

### 3.5. Contextual Regulation of mTORC1 by Sestrin2

**Figure 6:**
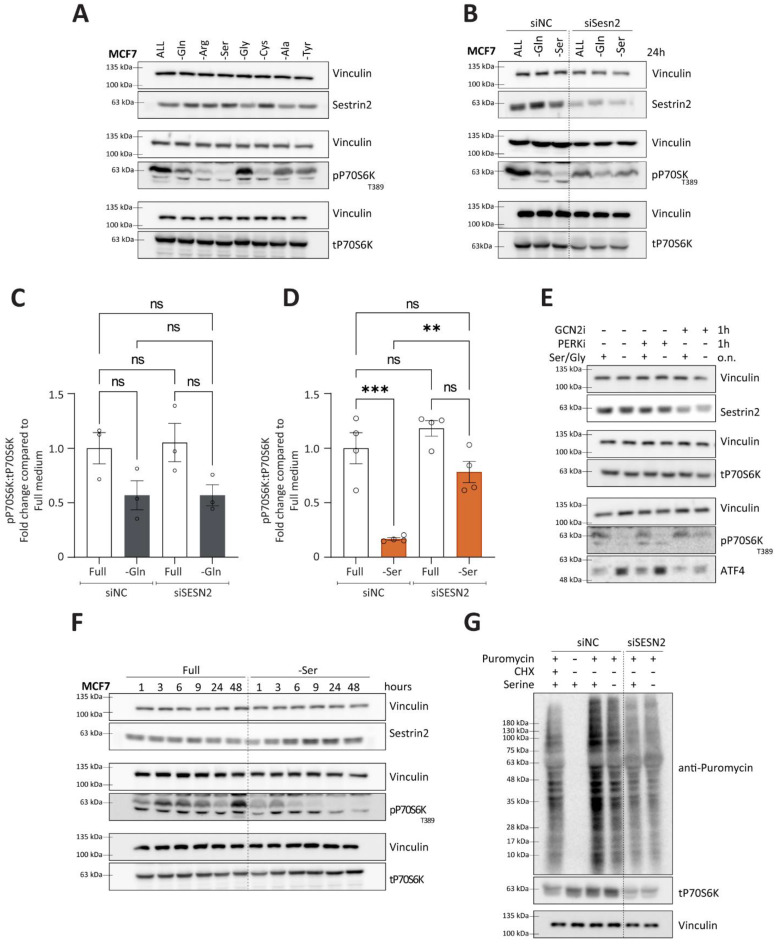
Sestrin2dependent regulation of mTORC1 upon amino acid deprivation. (**A**) Sestrin2 protein levels and phosphorylation of P70S6K after two days of amino acid deprivations; (**B**) phosphorylation of P70S6K upon SESN2 silencing in the presence or absence of indicated amino acids; (**C**,**D**) densitometric quantification of Western blots from three independent experiments in MCF7 cells (n = 3). ANOVA with a post hoc Tukey test was used to measure statistical significance; (**E**) Sestrin2, ATF4 levels, and the phosphorylation of P70S6K in MCF7 cells upon the cotreatment with GCN2 inhibitor (1 µM) and PERK inhibitor (1 µM) in either the presence or absence of serine (0.4 mM), glycine (0.4 mM), or in combination; (**F**) timedependent regulation of Sestrin2 and phosphorylation of P70S6K upon the serine deprivation; (**G**) protein synthesis in MCF7 cells measured with puromycin (90 µM) with CHX (20 µM) as a positive control upon siNC or siSESN2. Samples were re-probed for the vinculin control. Dots represent individual values. ** and *** indicate *p* ≤ 0.01 and 0.001, respectively. n.s. indicates statistical insignificance.

Nutritional stress, one of the regulators of Sestrin2, has been shown to play a role in modulating mTORC1 activity. Considering context-dependent regulation of Sestrin2, we sought to further investigate the relationship between Sestrin2 and mTORC1 in the context of a single non-essential amino acid deprivation. As expected, amino acid deprivation caused a reduction in phosphorylation of P70S6K (T389), a downstream effector of mTORC1, indicating impaired mTORC1 activity (Figure 6A). The degree of mTORC1 inhibition depended on the amino acid deprived. In MCF7, serine auxotroph cells, serine deprivation caused the most pronounced reduction in phospho-P70S6K levels (Figure 6A). Interestingly, mTORC1 inhibition was not always accompanied with Sestrin2 induction (i.e., alanine or tyrosine deprivations), suggesting Sestrin2-independent mechanisms of mTORC1 regulation. 

Hence, we sought to identify conditions that regulate mTORC1 in a Sestrin2-dependent manner. We silenced SESN2 and investigated phosphorylation of P70S6K upon glutamine or serine deprivation. As shown in Figure 6B,C, in group 1, characterized by a complex cellular response to the amino acid deprivations, SESN2 had no impact on mTORC1 activity. However, in group 2, where solely nutritional stress regulated Sestrin2, Sestrin2 became essential in blunting mTORC1 activity, measured by phosphorylation of P70S6K (Figure 6B,D). Solely serine, or combined serine and glycine deprivation inhibited mTORC1 activity in the Sestrin2-dependent manner (Figure S6A). 

Furthermore, an enhanced phosphorylation of AMPKα (T172) upon glutamine deprivation (supporting the claim of general metabolic reprogramming in the group 1) was independent of Sestrin2 (Figure S6B). In group 2, serine deprivation was inefficient in activating AMPKα, confirming solely nutritional stress upon serine removal (Figure S6B). These data indicate that SESN2 is dispensable in the cellular response to an energy crisis caused by amino acid deprivation (at least to the energy crisis signaling depicted through AMPK levels).

Additionally, inhibition of GCN2, and not PERK, rescued ATF4 levels and mTORC1 activity (Figure 6E). Furthermore, temporal investigation of phosphorylation of P70S6K and Sestrin2 revealed that the strong inhibition of the mTORC1 signaling pathway was accompanied with Sestrin2 induction shortly after serine (Figure 6F) or serine/glycine removal (Figure S5C). Finally, Sestrin2 was indispensable for the reduction of the protein synthesis upon serine removal in MCF7 (Figure 6G and Figure S6D). It is worth mentioning that silencing SESN2 in MCF7 cells reduced cellular survival (Figure S2D), probably resulting in reduced overall protein synthesis. Therefore, the puromycin incorporation is reduced upon SESN2 knock-down. 

Altogether, these data confirm the ATF4-Sestrin2-mediated inhibition of mTORC1, but solely in the absence of serine, in MFC7 cells, due to reduced serine biosynthesis rate and, hence, reduced intracellular serine levels. On the other hand, Sestrin2 was dispensable for mTORC1 regulation upon glutamine deprivation that caused overall cellular stress: oxidative, nutritional, and energetic stress. 

## 4. Discussion

Altered metabolism is a hallmark of highly proliferative cancerous cells, hence targeting cancer metabolism became an attractive anti-cancer strategy [6,30,31]. However, cellular metabolic plasticity contributes to resistance to metabolic modulators [32,33,34,35]. The goal of the present project was to elucidate regulation and the role of Sestrin2 during an adaptive cellular response to a single amino acid starvation.

Based on our data, obtained from three cancer cell lines derived from different tissue origins and representing different genetic backgrounds, we grouped non-essential amino acid deprivations based on their effects on cellular proliferation and Sestrin2 expression, and characterized the overall cellular response in the respective group. Group 1 consists of amino acids that uniformly impaired cellular proliferation and increased Sestrin2 expression in all three investigated cell lines. Additionally, our data support the literature to show that glutamine, arginine, and cysteine deprivations are accompanied with oxidative and energetic stress [36,37,38,39,40]. However, we showed that besides canonical nutritional stress, conducted through GCN2-ATF4 signaling pathway [20,41,42], oxidative stress was contributing to the Sestrin2 induction. Group 2 is a prime example of the negative correlation between Sestrin2 expression and cellular survival dependent on the cellular metabolic background. In PHGDH deficient cells, such as MCF7 [26], Sestrin2 levels were increased and proliferation was impaired upon the deprivation. More importantly, the upregulation of Sestrin2 was driven solely by nutritional stress through the GCN2-ATF4 signaling pathway. Group 3 represents outliers, that induced Sestrin2 without changing proliferation. We noticed that the Sestrin2 regulation in these conditions were driven solely through oxidative stress. Therefore, we have clearly shown a context-dependent regulation of Sestrin2 upon removal of a single amino acid, where the secondary effects of metabolic reprogramming, namely, oxidative or energetic stress, could also contribute to the Sestrin2 upregulation. 

Furthermore, many studies have shown Sestrin2-dependent mTORC1 inhibition and it has been reported that Sestrin2 upregulation upon leucine, isoleucine, lysine, glutamine, or arginine deprivation inhibits mTORC1 [20,22]. Nevertheless, in our experimental setup Sestrin2 was dispensable upon glutamine removal, but was essential in serine-deprived MCF7 cells to regulate mTORC1. The complex cellular response observed upon glutamine starvation suggests a complementary regulation of mTORC1 through several pathways [43]. In contrast, direct leucine sensing of Sestrin2 could be a reason for a more selective Sestrin2-dependent inhibition of mTORC1 for this branch of amino acid utilization/metabolism [21,44,45]. Glutamine deprivation causes leucine accumulation in glutamine-deprived pancreatic cancer cells [35], which could disrupt the Sestrin2:GATOR2 complex important for Sestrin2-dependent mTORC1 inhibition. It is important to note that in MCF7 cells, glutamine deprivation caused partial, while serine deprivation caused complete mTORC1 inhibition. Furthermore, non-essential amino acid deprivations are classified based on the experiments performed with three representative cell lines, namely, HCT116, HepG2, and MCF7. Thus, with the classification made here, we like to describe and highlight the complex and context-dependent regulation of Sestrin2 demonstrated in our experiments and by others, but not an ultimate classification of cellular response to amino acid deprivation, due to metabolic differences between cell lines [46]. Nevertheless, our results clearly support the notion that amino acid metabolism and sensing could be exploited to optimize cancer therapy [47,48]. The complexity of metabolic interactions clearly warrants further detailed analysis of genetic alterations (mutation, amplification, and deletion) linked to amino acid metabolism and sensing to discover specific dependencies of cancer cells for different amino acids and could possibly further elucidate the role of Sestrin2 in cancer metabolism.

## 5. Conclusions

We show for the first time that amino acid scarcity regulates mTORC1 activity through Sestrin2 only in full auxotroph cells, when the amino acid cannot be restored by metabolic reprogramming. Any metabolic changes needed for amino acid production, leads to partial replenishment, and also to the other sources of stress that regulate Sestrin2 and mTORC1 through independent pathways. Therefore, we propose that Sestrin2 could serve as an indicator of an underlying metabolic response to amino acid starvation which may, but not exclusively, result in cellular proliferation impairment. While Sestrin2 could be utilized for an initial metabolic profiling of each cell line to better understand the overall cellular response to amino acid starvation, there is still a need to further investigate how contextual regulation of Sestrin2 impacts the role of Sestrin2 to make more rational and personalized anti-tumor therapy.

## Data Availability

Not applicable.

## Supplementary Materials

The following supporting information can be downloaded at: https://www.mdpi.com/article/10.3390/cells11233863/s1, Figure S1: Basal PHGDH levels in various cell lines; Figure S2: Sestrin2 regulation and proliferation rate upon amino acid deprivations; Figure S3: Group 1 of amino acid deprivations causes metabolic, oxidative, and nutritional stress; Figure S4: Group 2 of amino acid deprivations causes solely nutritional stress; Figure S5: Group 3 of amino acid deprivations causes solely oxidative stress; Figure S6: Sestrin2-dependent regulation of mTORC1 upon amino acid deprivation.

## References

[1] Heiden M.G.V. (2011). Targeting cancer metabolism: A therapeutic window opens. Nat. Rev. Drug Discov..

[2] Xie H., Hanai J.-I., Ren J.-G., Kats L., Burgess K., Bhargava P., Signoretti S., Billiard J., Duffy K.J., Grant A. (2014). Targeting Lactate Dehydrogenase-A Inhibits Tumorigenesis and Tumor Progression in Mouse Models of Lung Cancer and Impacts Tumor-Initiating Cells. Cell Metab..

[3] Oshima N., Ishida R., Kishimoto S., Beebe K., Brender J.R., Yamamoto K., Urban D., Rai G., Johnson M.S., Benavides G. (2020). Dynamic Imaging of LDH Inhibition in Tumors Reveals Rapid In Vivo Metabolic Rewiring and Vulnerability to Combination Therapy. Cell Rep..

[4] Luengo A., Gui D.Y., Vander Heiden M.G. (2017). Targeting Metabolism for Cancer Therapy. Cell Chem. Biol..

[5] Lukey M.J., Katt W.P., Cerione R.A. (2017). Targeting amino acid metabolism for cancer therapy. Drug Discov. Today.

[6] Stine Z.E., Schug Z.T., Salvino J.M., Dang C.V. (2021). Targeting cancer metabolism in the era of precision oncology. Nat. Rev. Drug Discov..

[7] Liu G.Y., Sabatini D.M. (2020). Mtor at the Nexus of Nutrition, Growth, Ageing and Disease. Nat. Rev. Mol. Cell Biol..

[8] Saxton R.A., Sabatini D.M. (2017). Mtor Signaling in Growth, Metabolism, and Disease. Cell.

[9] Mossmann D., Park S., Hall M.N. (2018). mTOR signalling and cellular metabolism are mutual determinants in cancer. Nat. Rev. Cancer.

[10] Simcox J., Lamming D.W. (2022). The central moTOR of metabolism. Dev. Cell.

[11] Momcilovic M., Bailey S.T., Lee J.T., Fishbein M.C., Braas D., Go J., Graeber T.G., Parlati F., Demo S., Li R. (2018). The GSK3 Signaling Axis Regulates Adaptive Glutamine Metabolism in Lung Squamous Cell Carcinoma. Cancer Cell.

[12] Budanov A.V., Karin M. (2008). p53 Target Genes Sestrin1 and Sestrin2 Connect Genotoxic Stress and mTOR Signaling. Cell.

[13] Lee J.H., Cho U.S., Karin M. (2016). Sestrin Regulation of Torc1: Is Sestrin a Leucine Sensor?. Sci. Signal..

[14] Velasco-Miguel S., Buckbinder L., Jean P., Gelbert L., Talbott R., Laidlaw J., Seizinger B., Kley N. (1999). PA26, a novel target of the p53 tumor suppressor and member of the GADD family of DNA damage and growth arrest inducible genes. Oncogene.

[15] Peeters H., Debeer P., Bairoch A., Wilquet V., Huysmans C., Parthoens E., Fryns J.P., Gewillig M., Nakamura Y., Niikawa N. (2003). PA26 is a candidate gene for heterotaxia in humans: Identification of a novel PA26-related gene family in human and mouse. Hum. Genet..

[16] Budanov A.V., Shoshani T., Faerman A., Zelin E., Kamer I., Kalinski H., Gorodin S., Fishman A., Chajut A., Einat P. (2002). Identification of a novel stress-responsive gene Hi95 involved in regulation of cell viability. Oncogene.

[17] Ben-Sahra I., Dirat B., Laurent K., Puissant A., Auberger P., Budanov A., Tanti J.-F., Bost F. (2012). Sestrin2 integrates Akt and mTOR signaling to protect cells against energetic stress-induced death. Cell Death Differ..

[18] Lee J.H., Budanov A.V., Karin M. (2013). Sestrins Orchestrate Cellular Metabolism to Attenuate Aging. Cell Metab..

[19] Park H.-W., Park H., Ro S.-H., Jang I., Semple I.A., Kim D.N., Kim M., Nam M., Zhang D., Yin L. (2014). Hepatoprotective role of Sestrin2 against chronic ER stress. Nat. Commun..

[20] Ye J., Palm W., Peng M., King B., Lindsten T., Li M.O., Koumenis C., Thompson C.B. (2015). GCN2 sustains mTORC1 suppression upon amino acid deprivation by inducing Sestrin2. Genes Dev..

[21] Wolfson R.L., Chantranupong L., Saxton R.A., Shen K., Scaria S.M., Cantor J.R., Sabatini D.M. (2016). Sestrin2 is a leucine sensor for the mTORC1 pathway. Science.

[22] Byun J.-K., Choi Y.-K., Kim J.-H., Jeong J.Y., Jeon H.-J., Kim M.-K., Hwang I., Lee S.-Y., Lee Y.M., Lee I.-K. (2017). A Positive Feedback Loop between Sestrin2 and mTORC2 Is Required for the Survival of Glutamine-Depleted Lung Cancer Cells. Cell Rep..

[23] Bunz F., Dutriaux A., Lengauer C., Waldman T., Zhou S., Brown J.P., Sedivy J.M., Kinzler K.W., Vogelstein B. (1998). Requirement for P53 and P21 to Sustain G_2_ Arrest after DNA Damage. Science.

[24] Lochead J., Schessner J., Werner T., Wölfl S. (2015). Time-Resolved Cell Culture Assay Analyser (TReCCA Analyser) for the Analysis of On-Line Data: Data Integration—Sensor Correction—Time-Resolved IC50 Determination. PLoS ONE.

[25] Weger B.D., Weger M., Goerling B., Schink A., Gobet C., Keime C., Poschet G., Jost B., Krone N., Hell R. (2016). Extensive Regulation of Diurnal Transcription and Metabolism by Glucocorticoids. PLoS Genet..

[26] Chen J., Chung F., Yang G., Pu M., Gao H., Jiang W., Yin H., Capka V., Kasibhatla S., Laffitte B. (2013). Phosphoglycerate dehydrogenase is dispensable for breast tumor maintenance and growth. Oncotarget.

[27] Possemato R., Marks K.M., Shaul Y.D., Pacold M.E., Kim D., Birsoy K., Sethumadhavan S., Woo H.-K., Jang H.G., Jha A.K. (2011). Functional genomics reveal that the serine synthesis pathway is essential in breast cancer. Nature.

[28] Canovas B., Nebreda A.R. (2021). Diversity and versatility of p38 kinase signalling in health and disease. Nat. Rev. Mol. Cell Biol..

[29] Tajan M., Hennequart M., Cheung E.C., Zani F., Hock A.K., Legrave N., Maddocks O.D.K., Ridgway R.A., Athineos D., Suárez-Bonnet A. (2021). Serine synthesis pathway inhibition cooperates with dietary serine and glycine limitation for cancer therapy. Nat. Commun..

[30] Almouhanna F., Blagojevic B., Can S., Ghanem A., Wölfl S. (2021). Pharmacological activation of pyruvate kinase M2 reprograms glycolysis leading to TXNIP depletion and AMPK activation in breast cancer cells. Cancer Metab..

[31] Abu el Maaty M.A., Dabiri Y., Almouhanna F., Blagojevic B., Theobald J., Büttner M., Wölfl S. (2018). Activation of pro-survival metabolic networks by 1,25(OH)2D3 does not hamper the sensitivity of breast cancer cells to chemotherapeutics. Cancer Metab..

[32] Biancur D.E., Kimmelman A.C. (2018). The plasticity of pancreatic cancer metabolism in tumor progression and therapeutic resistance. Biochim. et Biophys. Acta (BBA) Rev. Cancer.

[33] Boudreau A., Purkey H.E., Hitz A., Robarge K., Peterson D., Labadie S., Kwong M., Hong R., Gao M., Del Nagro C. (2016). Metabolic plasticity underpins innate and acquired resistance to LDHA inhibition. Nat. Chem. Biol..

[34] Fendt S.-M., Frezza C., Erez A. (2020). Targeting Metabolic Plasticity and Flexibility Dynamics for Cancer Therapy. Cancer Discov..

[35] Tsai P.-Y., Lee M.-S., Jadhav U., Naqvi I., Madha S., Adler A., Mistry M., Naumenko S., Lewis C.A., Hitchcock D.S. (2021). Adaptation of pancreatic cancer cells to nutrient deprivation is reversible and requires glutamine synthetase stabilization by mTORC1. Proc. Natl. Acad. Sci. USA.

[36] Gwangwa M.V., Joubert A.M., Visagie M.H. (2019). Effects of glutamine deprivation on oxidative stress and cell survival in breast cell lines. Biol. Res..

[37] Polat I.H., Tarrado-Castellarnau M., Benito A., Hernandez-Carro C., Centelles J., Marin S., Cascante M. (2021). Glutamine Modulates Expression and Function of Glucose 6-Phosphate Dehydrogenase via NRF2 in Colon Cancer Cells. Antioxidants.

[38] Chen C.-L., Hsu S.-C., Chung T.-Y., Chu C.-Y., Wang H.-J., Hsiao P.-W., Yeh S.-D., Ann D.K., Yen Y., Kung H.-J. (2021). Arginine is an epigenetic regulator targeting TEAD4 to modulate OXPHOS in prostate cancer cells. Nat. Commun..

[39] Qiu F., Chen Y.-R., Liu X., Chu C.-Y., Shen L.-J., Xu J., Gaur S., Forman H.J., Zhang H., Zheng S. (2014). Arginine Starvation Impairs Mitochondrial Respiratory Function in ASS1-Deficient Breast Cancer Cells. Sci. Signal..

[40] Alborzinia H., Flórez A.F., Kreth S., Brückner L.M., Yildiz U., Gartlgruber M., Odoni D.I., Poschet G., Garbowicz K., Shao C. (2022). MYCN mediates cysteine addiction and sensitizes neuroblastoma to ferroptosis. Nat. Cancer.

[41] Ye J., Kumanova M., Hart L.S., Sloane K., Zhang H., De Panis D.N., Bobrovnikova-Marjon E., Diehl J.A., Ron D., Koumenis C. (2010). The GCN2-ATF4 pathway is critical for tumour cell survival and proliferation in response to nutrient deprivation. EMBO J..

[42] Sawa R., Ohnishi A., Ohno M., Nagata M., Wake I., Okimura Y. (2022). Specific amino acids regulate Sestrin2 mRNA and protein levels in an ATF4-dependent manner in C2C12 myocytes. Biochim. et Biophys. Acta (BBA) Gen. Subj..

[43] Yuneva M., Zamboni N., Oefner P., Sachidanandam R., Lazebnik Y. (2007). Deficiency in glutamine but not glucose induces MYC-dependent apoptosis in human cells. J. Cell Biol..

[44] Cangelosi A.L., Puszynska A.M., Roberts J.M., Armani A., Nguyen T.P., Spinelli J.B., Kunchok T., Wang B., Chan S.H., Lewis C.A. (2022). Zonated Leucine Sensing by Sestrin-Mtorc1 in the Liver Controls the Response to Dietary Leucine. Science.

[45] Gu X., Jouandin P., Lalgudi P.V., Binari R., Valenstein M.L., Reid M.A., Allen A.E., Kamitaki N., Locasale J.W., Perrimon N. (2022). Sestrin mediates detection of and adaptation to low-leucine diets in Drosophila. Nature.

[46] Nwosu Z.C., Megger D.A., Hammad S., Sitek B., Roessler S., Ebert M.P., Meyer C., Dooley S. (2017). Identification of the Consistently Altered Metabolic Targets in Human Hepatocellular Carcinoma. Cell. Mol. Gastroenterol. Hepatol..

[47] Butler M., van der Meer L.T., van Leeuwen F.N. (2021). Amino Acid Depletion Therapies: Starving Cancer Cells to Death. Trends Endocrinol. Metab..

[48] Gao S., Dai Z., Xu H., Lai L. (2022). Pinpointing Cancer Sub-Type Specific Metabolic Tasks Facilitates Identification of Anti-cancer Targets. Front. Med..

