# Supplementary material for: Cell Type-Specific Metabolic Response to Amino Acid Starvation Dictates the Role of Sestrin2 in Regulation of mTORC1
Source: Cells. 2022 Nov 30;11(23):3863. doi: 10.3390/cells11233863 (PMC9736985; doi:10.3390/cells11233863)
Supplement: Supplementary file 1 [file cells-11-03863-s001.zip › cells-1922430-supplementary.pdf]

Article

Cell Type-Specific Metabolic Response to Amino Acid Starvation Dictates the Role of Sestrin2 in Regulation of mTORC1

Biljana Blagojevic [1], Fadi Almouhanna [1,†], Gernot Poschet [2] and Stefan Wölfl [1,*]

[1] Institute of Pharmacy and Molecular Biotechnology, Heidelberg University, Im Neuenheimer Feld 364, 69120 Heidelberg, Germany;

[2] Metabolomics Core Technology Platform, Centre for Organismal Studies, Heidelberg University, Im Neuen-
heimer Feld 360,
69120 Heidelberg, Germany

* Correspondence: wolfl@uni-hd.de; Tel.: +49-6221-5448-80

† Present address: Institute Physiology and Pathophysiology, Heidelberg University, Im Neuenheimer Feld 326, 69120, Heidelberg, Germany.

Supplementary Materials: The following supporting information can be downloaded at: www.mdpi.com/xxx/s1, Figure S1: Basal PHGDH levels in various cell lines; Figure S2: Sestrin2 regulation and proliferation rate upon amino acid deprivations; Figure S3: Group 1 of amino acid deprivations causes metabolic, oxidative, and nutritional stress; Figure S4: Group 2 of amino acid deprivations causes solely nutritional stress; Figure S5: Group 3 of amino acid deprivations causes solely nutritional stress; Figure S6: Sestrin2-dependent regulation of mTORC1 upon amino acid deprivation.

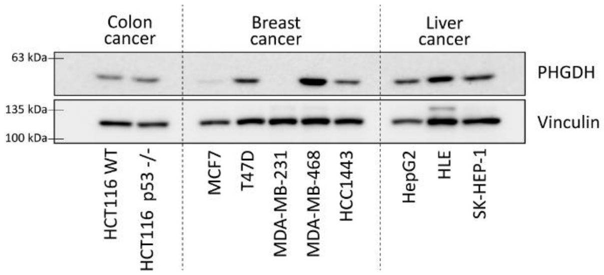

Figure S1. Basal PHDGH levels in various cell lines.

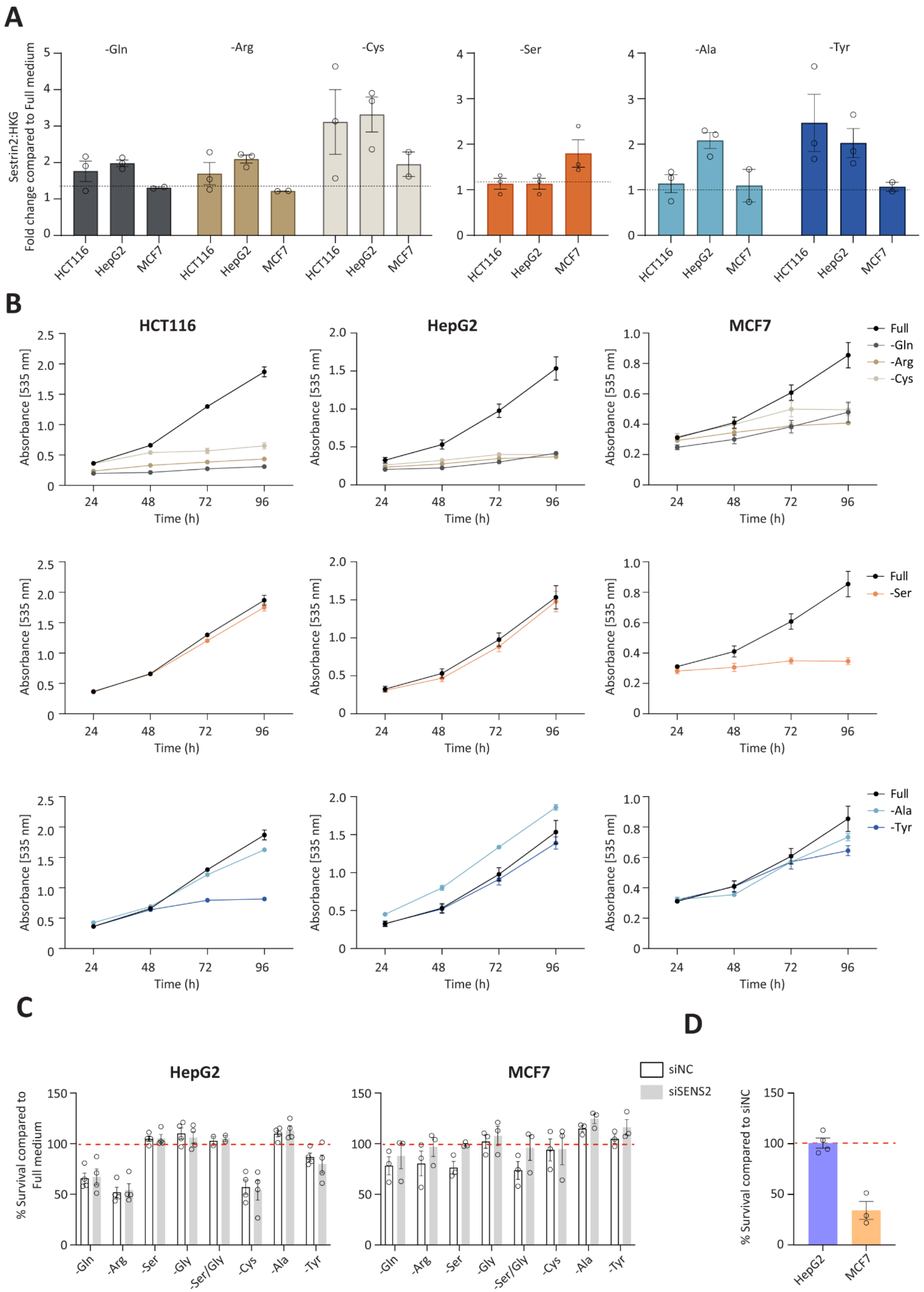

A
Sestrin2:HKG
Fold change compared to Full medium
-Gln
-Arg
-Cys
-Ser
-Ala
-Tyr
HCT116
HepG2
MCF7
B
HCT116
HepG2
MCF7
Absorbance [535 nm]
Time (h)
Full
-Gln
-Arg
-Cys
-Ser
-Ala
-Tyr
C
HepG2
MCF7
% Survival compared to Full medium
siNC
siSENS2
-Gln
-Arg
-Ser
-Gly
-Ser/Gly
-Cys
-Ala
-Tyr
D
% Survival compared to siNC
HepG2
MCF7

Figure S2. Sestrin2 regulation and proliferation rate upon amino acid deprivations. (**A**) Densitometric quantification of western blots from three independent experiments; (**B**) time-dependent survival measurements upon amino acid deprivations; (**C**) cellular survival in various media lacking an indicated amino acid(s) upon silencing SESN2; (**D**) cellular survival upon SESN2 knock-down compared to siNC. Dots represent individual values.

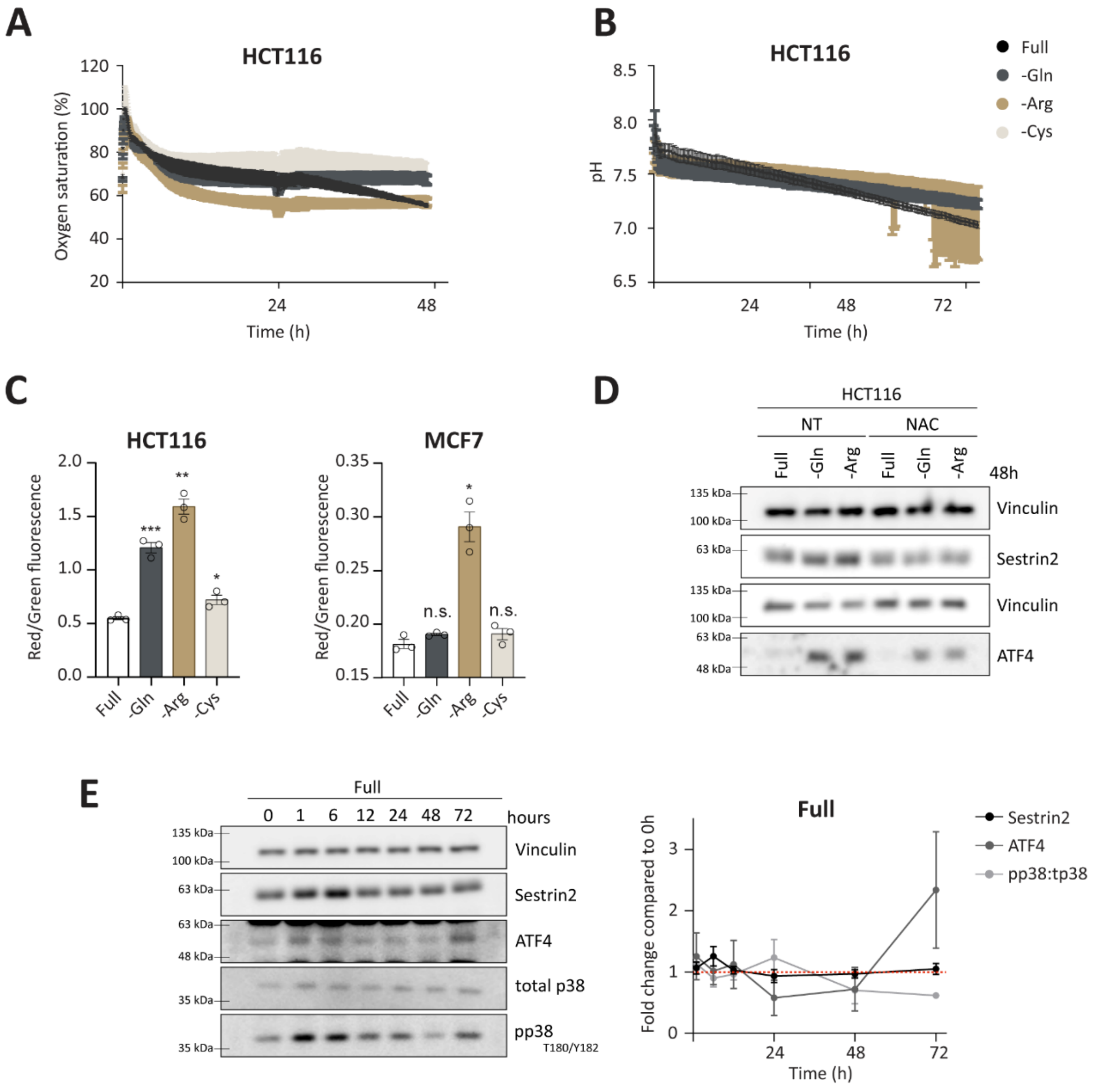

Figure S3. Group 1 of amino acid deprivations causes metabolic, oxidative, and nutritional stress. (**A-B**) Continuous measurements of oxygen saturation (**A**) and pH (**B**) values in extracellular medium; (**C**) mitochondrial membrane potential measured 48h of amino acid deprivations; (**D**) Sestrin2 and ATF4 levels upon 10 mM N-acetyl cysteine co-treatment; (**E**) time-dependent regulation of Sestrin2, ATF4, and phosphorylation of p38 in the full medium with densitometric quantification. Dots represent individual values. *, **, and *** indicate $p \leq 0.05$, 0.01, and 0.001, respectively.

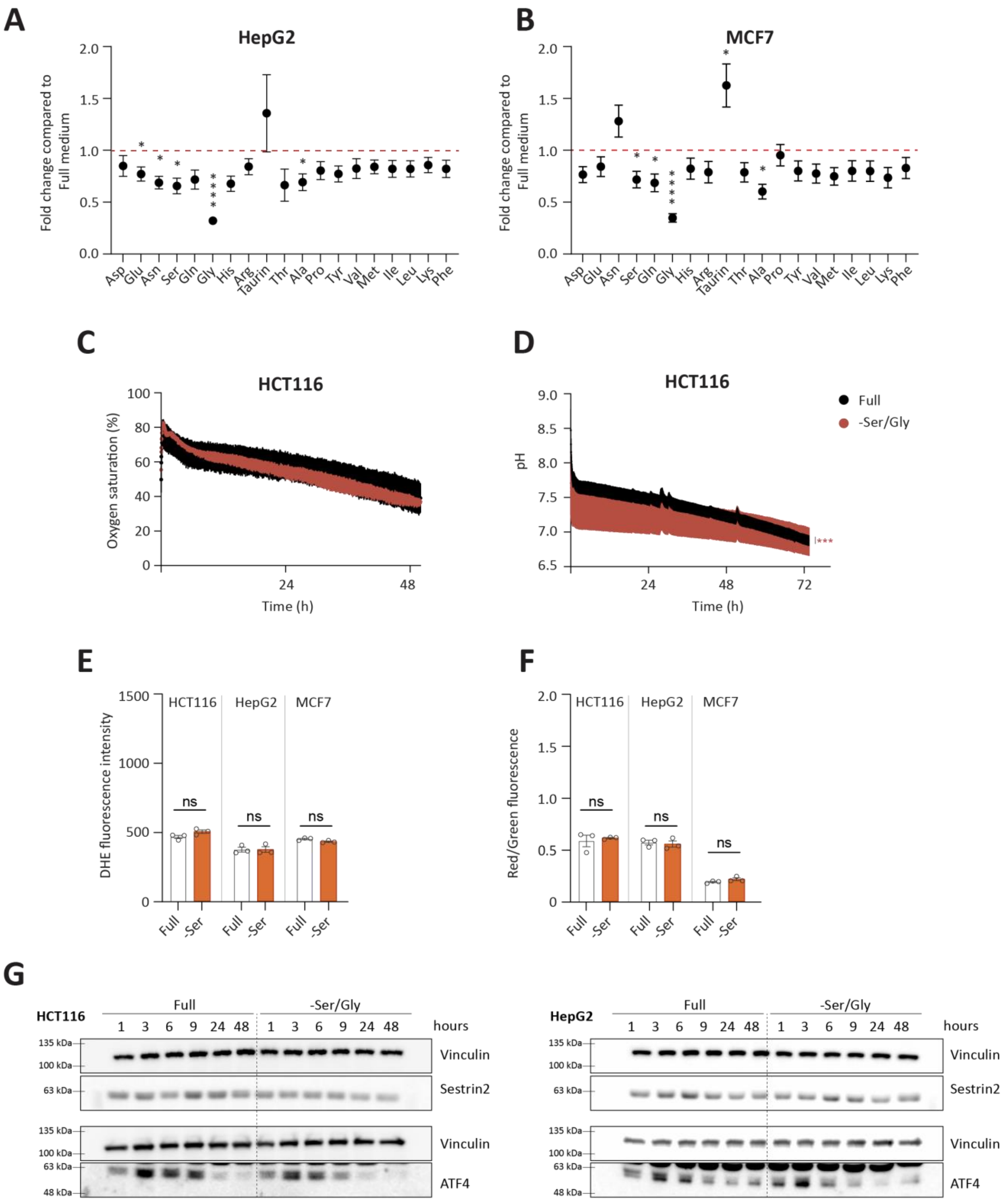

Figure S4. Group 2 of amino acid deprivations causes solely nutritional stress. (**A-B**) Intracellular amino acid levels upon 48h of serine/glycine deprivation in comparison to full medium. One-tailed t-test was used to measure statistical significance; (**C-D**) continuous measurements of oxygen saturation (**C**) and pH (**D**) values in extracellular medium; (**E-F**) intracellular ROS levels (**E**) and mitochondrial membrane potential (**F**) measured 48h of amino acid deprivations; (**G**) time-dependent regulation of Sestrin2 and ATF4 upon the serine/glycine deprivation. Dots represent individual values. * and **** indicate $p \leq 0.05$ and 0.0001, respectively. n.s. indicates statistical insignificance.

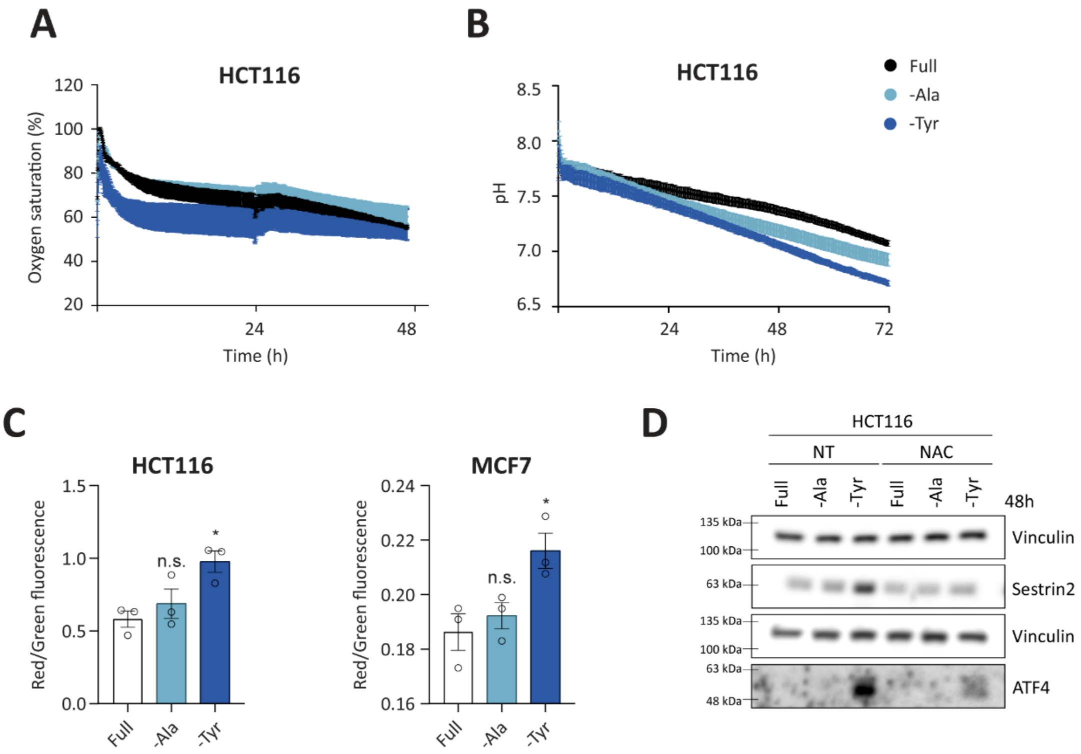

Figure S5. Group 3 of amino acid deprivations causes solely oxidative stress. (**A-B**) Continuous measurements of oxygen saturation (**A**) and pH (**B**) values in extracellular medium; (**C**) mitochondrial membrane potential measured 48h of amino acid deprivations; (**D**) Sestrin2 and ATF4 levels upon 10 mM N-acetyl cysteine co-treatment. Dots represent individual values. * indicates $p \leq 0.05$. n.s. indicates statistical insignificance.

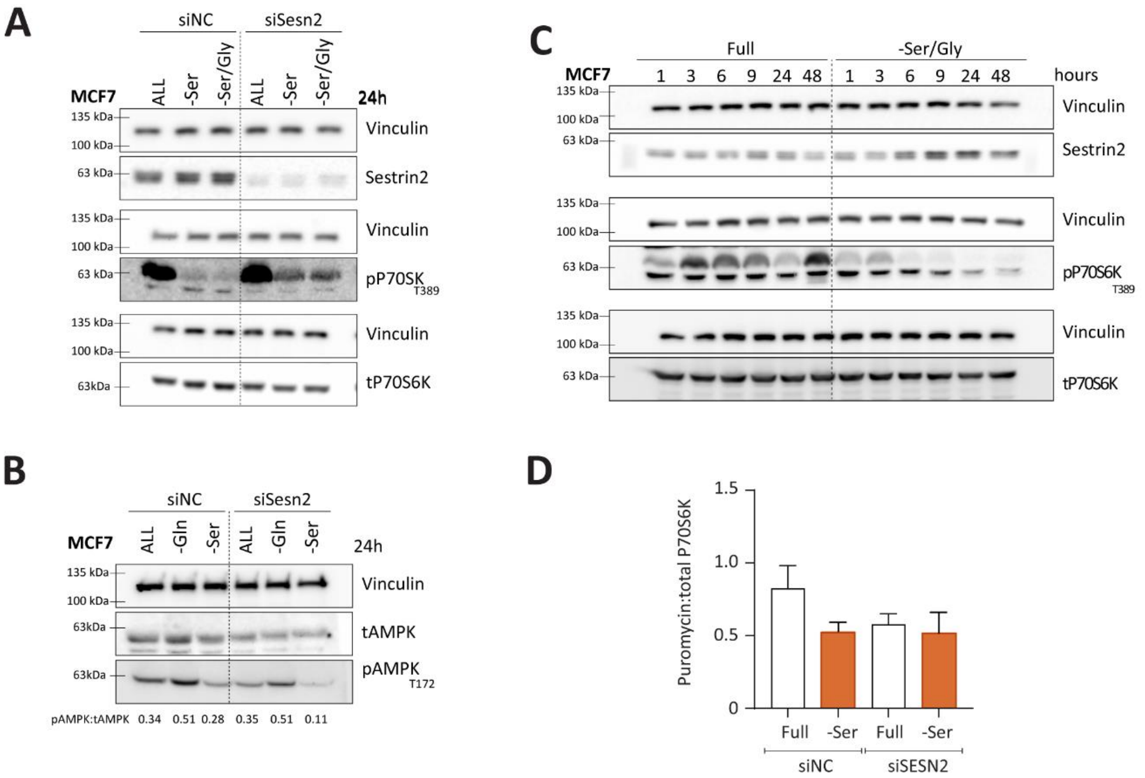

Figure S6. Sestrin2-dependent regulation of mTORC1 upon amino acid deprivation. (**A**) Phosphorylation of P70S6K upon SESN2 silencing in the presence or absence of indicated amino acids; (**B**) phosphorylation of AMPKα upon SESN2 silencing in the presence or absence of indicated amino acids; (**C**) time-dependent regulation of Sestrin2 and phosphorylation of P70S6K upon the serine/glycine deprivation; (**D**) densitometric quantification of puromycin experiment shown in Figure 6G (n = 2).
